# Pregnancy and Virologic Response to Antiretroviral Therapy in South Africa

**DOI:** 10.1371/journal.pone.0022778

**Published:** 2011-08-02

**Authors:** Daniel Westreich, Stephen R. Cole, Shashi Nagar, Mhairi Maskew, Charles van der Horst, Ian Sanne

**Affiliations:** 1 Department of Obstetrics and Gynecology, Duke Global Health Institute, Duke University, Durham, North Carolina, United States of America; 2 Department of Epidemiology, University of North Carolina-Chapel Hill, Chapel Hill, North Carolina, United States of America; 3 Clinical HIV Research Unit, University of the Witwatersrand, Johannesburg, South Africa; 4 Right to Care, Johannesburg, South Africa; 5 Division of Infectious Diseases, Department of Medicine, University of North Carolina-Chapel Hill, Chapel Hill, North Carolina, United States of America; University of Cape Town, South Africa

## Abstract

**Background:**

Although women of reproductive age are the largest group of HIV-infected individuals in sub-Saharan Africa, little is known about the impact of pregnancy on response to highly active antiretroviral therapy (HAART) in that setting. We examined the effect of incident pregnancy after HAART initiation on virologic response to HAART.

**Methods and Findings:**

We evaluated a prospective clinical cohort of adult women who initiated HAART in Johannesburg, South Africa between 1 April 2004 and 30 September 2009, and followed up until an event, death, transfer, drop-out, or administrative end of follow-up on 31 March 2010. Women over age 45 and women who were pregnant at HAART initiation were excluded from the study; final sample size for analysis was 5,494 women. Main exposure was incident pregnancy, experienced by 541 women; main outcome was virologic failure, defined as a failure to suppress virus to ≤400 copies/ml by six months or virologic rebound >400 copies/ml thereafter. We calculated adjusted hazard ratios using marginal structural Cox proportional hazards models and weighted lifetable analysis to calculate adjusted five-year risk differences. The weighted hazard ratio for the effect of pregnancy on time to virologic failure was 1.34 (95% confidence limit [CL] 1.02, 1.78). Sensitivity analyses generally confirmed these main results.

**Conclusions:**

Incident pregnancy after HAART initiation was associated with modest increases in both relative and absolute risks of virologic failure, although uncontrolled confounding cannot be ruled out. Nonetheless, these results reinforce that family planning is an essential part of care for HIV-positive women in sub-Saharan Africa. More work is needed to confirm these findings and to explore specific etiologic pathways by which such effects may operate.

## Introduction

The largest group of individuals living with HIV in Africa are women of child-bearing age [1]. In South Africa, young women have more than three times the estimated prevalence of HIV infection compared with young men [2], [3]. Furthermore, prevalence of HIV among pregnant women in South Africa was estimated at 28% in 2007, and may be as high as 40% among pregnant women ages 30–34: substantially higher than the overall adult prevalence [3]. In South Africa, antenatal testing is a key way in which women receive an HIV diagnosis; as such, pregnancy is a common indication for the initiation of highly active antiretroviral therapy (HAART) for the prevention of mother to child transmission [4]. Pregnancy is also common after clinically indicated initiation of HAART [5], [6].

Numerous studies have focused on optimal methods for prevention of mother to child transmission of HIV and subsequent response to HAART [7], [8], [9], [10], [11], [12], [13], as well as issues of fertility during HAART [14]. There is likewise a long history of studies examining the effect of pregnancy on HIV disease progression in the pre-HAART era [15], [16], [17], [18], [19] and a growing body of research on the impact of pregnancy on response to HAART in higher income countries [20], [21], [22], [23], [24]. However, to date there has been very little research examining effects of pregnancy on maternal response to HAART in sub-Saharan Africa [25]. This relative absence of evidence is striking given the unequivocal statement by the WHO that women's health should be “the overarching priority in decisions about ARV treatment during pregnancy” [26].

There are a number of reasons to hypothesize that response to HAART may be compromised during pregnancy. Pregnancy is associated with increases in blood volume and body mass index, which may lead to underdosing of drugs [27], [28]. Levels of cytochrome p450, and in particular CYP3A isoenzymes, may rise during pregnancy [29], increasing the metabolism of two antiretroviral drugs often given to HIV-positive pregnant women, lopinavir and nevirapine; thus pregnant women may experience reduced concentrations of both drugs [27], [29], [30], [31]. Additionally, beta-estradiol levels increase substantially in pregnancy [6], [32]; beta-estradiol may attenuate the efficacy of stavudine [32], another component of first-line HAART in South Africa. Last, social pressures related to pregnancy including stigma and fear of intimate partner violence [25], as well as responsibilities of new motherhood, may compromise adherence to HAART and thus virologic response to therapy. We thus hypothesized that pregnancy increases risk of virologic failure.

Much remains unknown about both short- and long-term risks associated with pregnancy in HIV-positive women receiving HAART, especially in sub-Saharan Africa. With large numbers of HIV-positive women becoming pregnant [14], it is vital to understand the impact of pregnancy on virologic outcomes of HAART.

## Materials and Methods

### Ethics statement

This research was based on de-identified previously collected clinical records, and was declared exempt from human subjects review by the University of the Witwatersrand, the University of North Carolina at Chapel Hill, and Duke University.

### Study population and design

We performed a prospective observational cohort study in the database of the Themba Lethu Clinic [33]. The Themba Lethu Clinic (henceforth, TLC) Cohort is a study of adults initiating HAART in Johannesburg, South Africa. The program is funded by the South African National and Gauteng Department of Health, with support from Right to Care and funding by USAID and PEPFAR. The TLC, sited at the regional Helen Joseph Hospital in urban Johannesburg, has over 17,000 patients in care and is the largest single clinic providing HAART in South Africa, and one of the largest HAART clinics worldwide. We studied previously antiretroviral therapy-naïve women from the time of HAART initiation between 1 April 2004 and 30 September 2009 at TLC. We followed these women until they experienced an outcome, administrative end of follow-up on 31 March 2010, or the end of care due to drop-out, death, or transfer of care to another site. Additional exclusions (by age and baseline pregnancy status) are described below.

Typical first-line HAART included stavudine, lamivudine, and efavirenz. Due to concerns about teratogenicity, women found to be pregnant are typically placed on the boosted protease inhibitor Kaletra (lopinavir and ritonavir) rather than efavirenz, while non-pregnant women with declared pregnancy intention at baseline are placed on nevirapine or Kaletra rather than efavirenz. Additional details of the TLC clinical database, clinic procedures, and outcomes have been described previously [33], [34], [35]. Here, we note that clinical data are captured prospectively in the TLC and that accuracy of data entry has been previously validated [33]. However, subjects did not receive care for pregnancy in the TLC, and thus data on fetal outcomes are not available.

### Definitions and data

The main exposure in this study was “ever became pregnant after HAART initiation”; that is, an incident pregnancy occurring subsequent to HAART initiation, regardless of length and outcome of the pregnancy. Pregnancy was defined based on clinical finding of pregnancy recorded in the clinical database in the course of standard HIV care. We concentrated on incident rather than prevalent pregnancy (i.e., pregnancy ongoing at the time of HAART initiation) due to concerns about confounding by indication. Women with prevalent pregnancy often initiate HAART *because they are pregnant*, and thus may be systematically different than women who initiate HAART for their own health and later become pregnant. Thus, women with prevalent pregnancy might be in general healthier than other women; this might manifest in a greater resiliency to drug toxicities or differing adherence to HAART which would obscure the effect of pregnancy itself. To avoid this bias, we excluded women who were prevalent at baseline from the main analysis, employing a “new pregnancy design” similar to the “new-user design” of pharmacoepidemiology [36]. Alternative exposure definitions were explored in sensitivity analysis.

The main outcome in this study was virologic failure, which was defined following Riddler et al. [37] as either a failure to achieve virologic suppression of plasma HIV-1 RNA to less than 400 copies/ml within six months of HAART initiation, or a viral rebound to above 400 copies/ml at any time after initial suppression. Confirmation of outcome by a second viral load test within 30 days was obtained when possible, but data from patients missing a confirmatory sample were included as failures [37]. In the main analysis, both death and drop-out were treated as censoring conditions; in sensitivity analysis we examined alternative outcomes and censoring variables.

Adherence to HAART was estimated from pharmacy records, as the time-updated cumulative proportion of days between pharmacy visits in which a subject had antiretroviral drugs available, included in regression models as a three-category variable (<95%, 95–99.9%, 100%). This estimate of adherence is an *upper limit* on potential adherence, because drugs can only be taken correctly if they are available.

### Statistical analysis

As noted above, the main analysis focused on the effect of incident pregnancy on time to virologic failure. A key concern of this analysis was the possibility of time-varying confounding affected by prior exposure [38], [39]. Thus, in addition to time-updated adjusted Cox models, we also used marginal structural Cox proportional hazards models [38], [39] to estimate hazard ratios (HRs), as well as confounding-adjusted extended Kaplan-Meier curves [40]. In both these cases, inverse probability weights were used to account for bias due to both confounding [41] and drop-out [38]. In the main analysis, weights were truncated at the 1^st^ and 99^th^ percentile to reduce the variance of estimates [42]. We used crude Kaplan-Meier curves to estimate crude five-year risk differences with confidence intervals derived from 200 bootstraps of the data.

In all multivariable analyses, we considered the following confounders of the effect of pregnancy on time to virologic failure, based on previous literature and biological mechanism. Confounders measured at baseline (HAART initiation) included age, ethnicity, employment status, current tuberculosis, calendar date of HAART initiation, history of smoking, and WHO stage. Confounders measured over time included weight, body mass index, hemoglobin, CD4 count and CD4 percent, antiretroviral drug regimen, and drug adherence. We used restricted cubic splines to flexibly control for age, body mass index, CD4 count, and time-on-study. Drug regimen was lagged by three months to ensure that we controlled for drug changes *preceding* pregnancy, rather than changes in drug regimen *affected by* pregnancy. We did not control for baseline viral load because it is collected in less than 25% of participants.

### Sensitivity analysis and missing data

To test analytic assumptions, we performed several sensitivity analyses in addition to the main analysis; these sensitivity analyses addressed issues in definitions of the population, exposure, and outcome, as well as technical decisions in the modeling.

We restricted the population to those women who had suppressed virus by six months after HAART initiation, and looked at subsequent incident pregnancy and virologic failure (sensitivity analysis 1). We considered a more inclusive definition of prevalent pregnancy for exclusion from the study population, based on initial drug regimen as well as database-recorded pregnancy status, to ensure that undercounting of prevalent pregnancy was not a source of bias (2). We examined the effect of pregnancy on less-specific outcomes, namely virologic failure or mortality (3), and virologic failure, mortality, or drop-out (4). We also examined a more restrictive outcome, in which we considered virologic failures *only* if they were confirmed within 30 days (5). We combined analyses 1 and 5, restricting to those with initial suppression who also had confirmed virologic failure (6). We considered an exposure of prevalent and incident pregnancy together, abandoning the “new-user” design described above (7). In all analyses, longitudinal data were carried forward from the most recent observed value. Missing data led to approximately 9% missing observations in the final analysis. The last two sensitivity analyses used a multiple imputation analysis to account for missing data (8), and in dropped carry-forward of time-updated variables (9).

### Role of the funding source

The funding sources had no involvement in the design or conduct of the study, in the collection, management, analysis, or interpretation of the data, in the preparation, writing, review or approval of this manuscript, or in the decision to submit this manuscript for publication.

## Results

The initial study population comprised 7,181 women at time of HAART initiation. After exploring univariate distributions of age by baseline pregnancy status, we excluded women who were over age 45 at baseline (1 pregnant woman, 1,138 non-pregnant women) to improve comparability and interpretability of final effect estimates [43]. This left 6,042 women; excluding 548 (9%) women who were pregnant at time of HAART initiation yielded a total sample size for the main analysis of 5,494 women of whom 541 experienced an incident pregnancy during follow-up. These 5,494 women contributed a total of approximately 11,600 person-years (139,272 person-months) to this analysis, of which 11,826 person-months (8.5%) were exposed, occurring coincident with or subsequent to an incident pregnancy. Median follow-up time in all women was 18 (IQR 9, 37) months.

**Figure 1:**
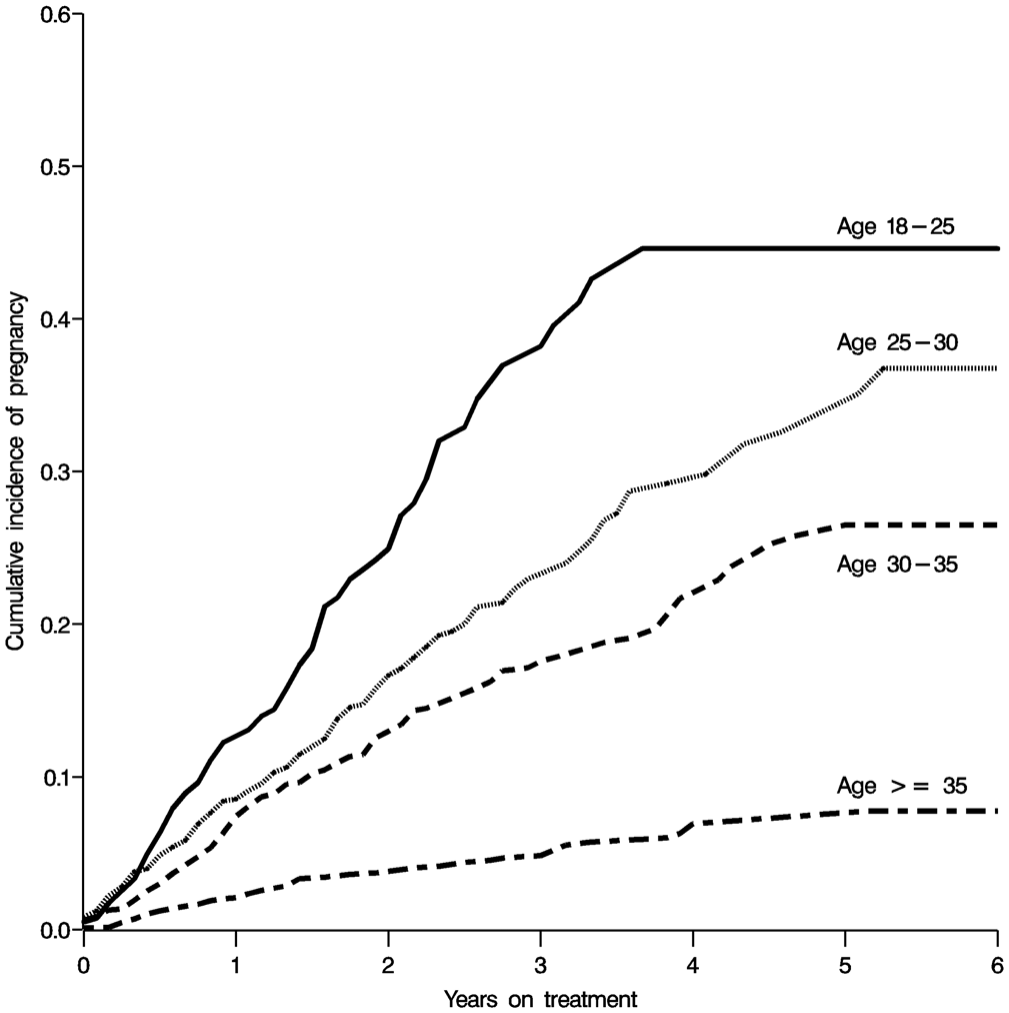
Crude cumulative incidence of pregnancy since date of HAART initiation, stratified by baseline age among 5,494 HIV-positive women initiating HAART in Johannesburg, South Africa from 1 April 2004 to 30 September 2009.

Baseline characteristics of the 5,494 subjects are given described in Table 1; in addition, Table 1 shows baseline and time-updated characteristics of the subset of 541 women who experienced an incident pregnancy. In general, at time of incident pregnancy, women were healthier than the average woman at time of HAART initiation, with a higher CD4 count, hemoglobin, and body mass index (BMI). At the time of incident pregnancy, the vast majority of women (94%) had a suppressed viral load. Age at HAART initiation was among the strongest predictors of both baseline and incidence pregnancy (Figure 1). Of note, an estimated 44% (95% confidence limits [CL] 36, 54%) of women who were 18–25 years old at the time of HAART initiation experience incident pregnancy within four years HAART initiation in this cohort.

**Table 1:** Characteristics of 5,494 women at time of HAART initiation, and a subpopulation of 541 of those women at time of incident pregnancy, in Johannesburg, South Africa from 1 April 2004 to 30 September 2009.

Demographics	At baseline(n = 5,494)	At incident pregnancy(n = 541)
Age *years*	34 (29, 38)	32 (28, 35)
African ethnicity	5,293 (96.3)	531 (98.2)*
Employed	2,233 (40.6)	216 (40.0)*
History of smoking	269 (4.9)	21 (3.9)*
Clinical		
HAART regimen		
d4t-3TC-EFV	4580 (83.4)	222 (41.0)
d4t-3TC-NVP	473 (8.6)	81 (15.0)
d4t-3TC-LPVr	146 (2.7)	116 (21.4)
Weight *kilograms*	57 (49, 65)	64 (56, 73)
Body mass index *kg/m^2^*	22.2 (19.5, 25.5)	24.8 (21.9, 27.7)
Body mass index category *kg/m^2^*		
<18.5	930 (17.7)	22 (4.1)
18.5–24.9	2,845 (54.2)	260 (48.3)
25.0–29.9	987 (18.8)	172 (32.0)
≥30	488 (9.3)	84 (15.6)
WHO stage III or IV	2,369 (43.1)	235 (43.4)*
Current tuberculosis	963 (17.5)	88 (16.3)*
Laboratory		
Hemoglobin, low‡	2,914 (54.7)	113 (21.1)
CD4 count *cells/mm^3^*	93 (35, 164)	304 (189, 433)
CD4 count category *cells/mm^3^*		
≤50	1,736 (32.6)	18 (3.3)
51–100	1,075 (20.2)	22 (4.1)
101–200	1,864 (35.0)	112 (20.8)
201–350	521 (9.8)	167 (31.0)
>350	133 (2.5)	220 (40.8)
Viral load† *log copies/ml*	4.2 (3.4, 4.6)	1.7 (1.7, 1.7)
Viral load category *log copies/ml*		
≤400	308 (20.0)	444 (93.9)
401–10000	251 (16.3)	10 (2.1)
>10000	984 (63.8)	19 (4.0)

d4T: stavudine. 3TC: lamivudine. EFV: efavirenz. NVP: nevirapine. LPVr: Lopinavir-ritonavir (Kaletra). Categorical variables are expressed as number (% of total non-missing); continuous variables are expressed as median (interquartile range).

*These measures are baseline measures, not updated to time of incident pregnancy.

‡ After adjustment for altitude, lower limit of normal hemoglobin is 11.35 g/dl.

† Baseline viral load was missing in 3951 (72%) women at baseline, and in 68 of women at time of exposure.

At baseline, 87% of women initiated a HAART regimen including efavirenz (83% were on the standard first-line HAART regimen of stavudine, lamivudine, and efavirenz), suggesting a lack of intention to become pregnant; 431 of these women later experienced a pregnancy. At the time of incident pregnancy, fewer women (53%) were receiving efavirenz. Many of these women switched to non-efavirenz-based HAART during pregnancy: 38% remained on efavirenz by month 3 of pregnancy. By month 5 of pregnancy, only 27% remained on efavirenz, the same proportion as remained on efavirenz at 9 months. By 18 months after conception, the proportion receiving efavirenz had risen back to 55%.

Of the 5,494 women considered in the main analysis, 81 women experienced virologic failure after an incident pregnancy (15% of the 541 exposed women); 748 (15%) of non-pregnant women experienced this outcome. Of the 829 total failures analyzed in main analysis, only 52 were confirmed within 30 days and 140 within 60 days. Examining all potential failures (not just those included in main analysis), 189 women had any virologic failure which was confirmed within 30 days. About one-third (n = 253) of total virologic failures were a failure to suppress within six months; the remaining two-thirds occurred after six months. Before experiencing the outcome or administrative censoring, 50 pregnant women (9%) died or dropped out, compared with 1680 (34%) non-pregnant women.

**Figure 2:**
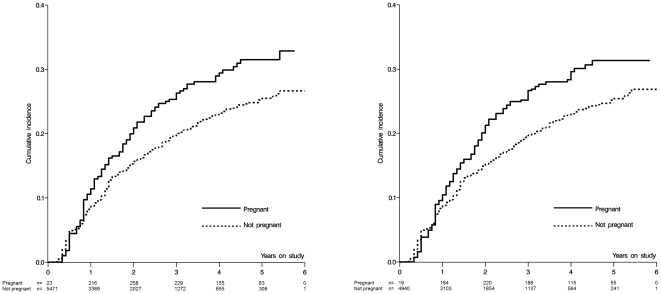
Crude and weighted cumulative incidence curves for the effect of pregnancy on time to virologic failure among 5,494 HIV-positive women initiating HAART in Johannesburg, South Africa from 1 April 2004 to 30 September 2009. Sample sizes in weighted curves are weighted sample sizes.

In the main analysis, the crude HR for the total effect of incident pregnancy on time to virologic failure over all of follow-up was 1.37 (95% CL 1.08, 1.73); the adjusted was 1.35 (95% CL 1.03, 1.77); and the weighted HR was 1.34 (95% CL 1.02, 1.78) (Table 2). The crude estimated absolute difference for the total effect of incident pregnancy on 5-year risk of virologic failure was 0.06 (−0.02, 0.14), an estimated 6% increase in virologic failure by five years. Figure 2 shows the crude and weighted extended Kaplan-Meier curves for the effect of pregnancy on time to virologic failure.

**Table 2:** Estimated effect of incident pregnancy on time to virologic failure among 5,494 women initiating HAART in South Africa, 2004–2009.

	No. of events	Person-months of follow-up	HR	95% CL
Unadjusted				
Not pregnant	748	127,446	1.	
Pregnant	81	11,826	1.37	1.08, 1.73
Adjusted†			1.35	1.03, 1.77
Weighted†				
Not pregnant	675‡	115,376	1.	
Pregnant	67‡	9,650	1.34	1.02, 1.78

HR, hazard ratio; CL, confidence limit.

† Both standard adjusted and weighted models accounted for the same set of covariates, namely age, ethnicity, history of smoking, employment status, active tuberculosis at study entry, calendar date at entry, WHO stage, and time-updated weight, body mass index, hemoglobin, CD4 count and percent, drug regimen, and drug adherence.

‡ Weighted event and person-month counts are given. Unweighted, these numbers are 688/116,884 and 69/9,918.

Among women who experienced a pregnancy, virologic failure was more common *during* pregnancy than *after* pregnancy; of the 81 failures observed, 42 took place during the nine months following reported start of pregnancy and 39 took place thereafter. A time-adjusted crude rate ratio comparing failure rate during the 9 months of the incident pregnancy exposure compared to other times was 1.49 (95% CL 1.09, 2.03), slightly stronger than the crude main effect estimate; likewise, the weighted rate ratio was slightly further from the null at 1.42 (95% CL 1.00, 2.01).

An additional, predictive (non-causal) analysis was undertaken to characterize the associations of baseline and time-updated variables on risk of the outcome (Table 3). To increase interpretability of these predictive factors, this analysis used less-flexible categorical parameterizations of BMI and CD4 count, and controlled for confounding only by time-updated measures of risk factors. As a result, the adjusted hazard ratio for pregnancy itself from this model was biased, and was therefore excluded from Table 3. This model showed that older age, baseline employment status, and lower BMI were associated with lower HR for virologic failure; and that lower CD4 count, use of nevirapine (compared with efavirenz), and less-than-perfect adherence were associated with higher HRs for virologic failure.

**Table 3:** Adjusted hazard ratios for non-causal associations of selected baseline and time-updated characteristics with time to virologic failure in 5,494 women initiating HAART in Johannesburg, South Africa from 1 April 2004 to 30 September 2009.

Time fixed characteristics	aHR	95% CL
Baseline age (effect of 5-year increase)	0.89	0.83, 0.94
Employed	0.81	0.70, 0.94
History of smoking	1.33	0.98, 1.79
Baseline tuberculosis	1.09	0.89, 1.33
WHO stage III or IV	1.11	0.95, 1.30
Time updated characteristics	aHR	95% CL
Body mass index category *kg/m^2^*		
<18.5	0.64	0.40, 1.03
18.5–24.9	0.71	0.52, 0.97
25.0–29.9	0.86	0.67, 1.11
≥30	1.	NA
Hemoglobin, low‡ *grams/dl*	1.01	0.85, 1.19
CD4 count category *cells/mm^3^*		
≤50	5.00	3.60, 6.96
51–100	5.35	3.98, 7.19
101–200	3.57	2.85, 4.49
201–350	2.08	1.69, 2.56
>350	1.	NA
Current drug regimen		
Efavirenz	1.	NA
Nevirapine	1.30	1.04, 1.62
Kaletra	1.17	0.91, 1.50
Adherence		
<95%	1.33	1.12, 1.57
95–99.9%	1.22	1.01, 1.46
100%	1.	NA

aHR, adjusted hazard ratio. Model controls additionally for incident pregnancy, time on study, calendar time, weight, and ethnicity.

‡ After adjustment for altitude, lower limit of normal hemoglobin is 11.35 g/dl.

Results from sensitivity analyses are summarized in Table S1. Restriction to women who had suppressed virus by six months (and who had not yet experienced pregnancy by that point) yielded a point estimate similar but less precise than the main effect: HR = 1.39 (95% CL 0.97, 1.99) (analysis 1, Table S1). The exclusions of additional women suspected of being pregnant at baseline likewise had little effect on the estimate of effect (analysis 2). In general, as the outcome and exposure became less strictly defined (analyses 3, 4, 7) the hazard ratio moved toward or to the null; most notably, when the outcome was failure, drop-out, or death, the point estimate was 0.96 (95% CL 0.78, 1.17). When outcome was *more* strictly defined, as any virologic failure confirmed within 30 days (analysis 5; starting with the 189 virologic failures described above), results were stronger but less precise (HR = 1.43, 95% CL 0.80, 2.55); results were similar when allowing a 60-day confirmation window. Restricting to women with both initial virologic suppression and confirmed virologic failure (analysis 6; total 101 outcomes) yielded an estimate somewhat closer to the null and much less precise, compared with the main result. The multiple imputation analysis (10 imputations, analysis 8) yielded a point estimate slightly closer to the null (HR = 1.27), as did dropping the “carry-forward” of time-updated variables (analysis 9, HR = 1.25).

## Discussion

In this observational study of HIV-positive women initiating HAART in South Africa, we found that incident pregnancy after HAART initiation is associated with increased relative and absolute risk of virologic failure of therapy during follow-up. Our main analysis estimated that pregnancy is associated with a hazard ratio of 1.34 (95% CL 1.02, 1.78), and an estimated absolute 6% increase in virologic failure by five years of follow-up.

Our results were stronger when the question being examined was more specific, and weaker when the question was more broad. In the former case, restricting to the period of pregnancy itself (rather than time after pregnancy) yielded an incidence rate ratio further from the null than main results; so did analyzing only confirmed virologic failures. In contrast, as the definitions of exposure and outcome used in the main analysis (incident pregnancy only as the exposure; virologic failure only as the outcome) grew less specific (for example, prevalent and incident pregnancy together as exposure; virologic failure and death together as the outcome), effects were generally closer to the null. Multiple imputation analysis suggested that missing data might bias these results slightly away from the null, but came to the same qualitative conclusion as the main analysis.

Despite substantial interest in issues of pregnancy and HIV disease progression, to date there has been almost no research performed *in populo* on the effect of pregnancy on virologic outcomes of HAART in sub-Saharan Africa, the setting where such findings are most important. We were able to find only two previous reports that addressed these issues outside of Africa. This first study, in the Swiss HIV Cohort, examined 372 pregnancies among 342 women who started HAART before and during pregnancy; compared to non-pregnant women, investigators found no increase in risk of virologic failure, and some indication of a reduction in risk [22]. A second study, in the United States, found high incidence of virologic rebound in the early post-partum period; however, this work examined only 63 women some of whom initiated HAART during, rather than before, pregnancy [44]. The generalizability of both studies to sub-Saharan Africa may be limited due to developed-world settings. The present study, involving over 11,000 person-years of follow-up in South Africa, helps fill a significant gap in the literature. Of course, the replication of this analysis in other sub-Saharan African cohorts is critical for confirming, or refuting, these findings.

Beyond large sample size, there were several key strengths of this study. Data were collected prospectively in a previously validated clinic database [33]. Issues of time-varying confounding affected by prior treatment were dealt with appropriately, using marginal structural Cox proportional hazards models [38] and weighted Kaplan-Meier curves [40]. Unlike in previous analyses [38], [45], a traditionally adjusted regression analysis did not provide markedly different answers than the marginal structural model. Nonetheless, the marginal structural model approach is preferred in situations where such bias may be possible; here, such bias was possible due to the effect of pregnancy on changes in antiretroviral therapy regimen, among other factors [46]. Last, previous reports from this cohort [33], [34] suggest that results from the Themba Lethu Clinic are generally comparable to other cohorts in sub-Saharan Africa; thus, we believe that the present results will have good generalizability to (at least) other urban HAART cohorts in sub-Saharan Africa.

With regard to generalizability, we made two key exclusions in our main analysis: women pregnant at baseline, and women over the age of 45. The former group was excluded because of concerns about confounding and potential selection bias; the latter because women older than 45 are very unlikely to become pregnant (that is, to become exposed) and thus present a relatively small group for whom inference is difficult due to problems of positivity [43]. The present findings should not be extrapolated to either of these groups without significant further study.

Other limitations of this study should be noted. We analyzed observational data from a clinical database, and thus uncontrolled confounding remains a possible threat to the validity of this study; for example, data on parity were missing for these subjects. Another potential confounder is baseline viral load, which was available in only 25% of women studied; however, restriction to women who had successfully suppressed virus by six months after HAART initiation confirmed the main analysis (sensitivity analysis 1), suggesting that this baseline viral load was not a critical source of bias in this study. Similarly, while we strove to exclude all women with a history of single-dose nevirapine exposure from our study, some such women may have been overlooked.

It is also possible that the observed increased rate of virologic failure after incident pregnancy is the consequence of detection bias: pregnant women may receive more lab tests than non-pregnant women. However, compared to non-pregnant women, the pregnant women were no more likely to have viral load measured (rate ratio for viral load testing comparing pregnant to non-pregnant person-time was 1.02, 95% CL 0.97, 1.08) and were less likely to have CD4 counts measured (rate ratio 0.85, 95% CL 0.81, 0.89). Thus, results are unlikely to be explained by detection bias.

Last, the interpretation of Table 3 deserves special caution. The main analysis attempted to obtain an estimate of the causal effect of pregnancy on time to virologic failure; but no such claims are made regarding Table 3. Table 3 explicitly represents a predictive, and not a causal, analysis.

In this study, we estimated the total effect of pregnancy on virologic failure, rather than direct or indirect effects that might comprise the total effect. However, estimation of total effects must be interpreted and generalized with caution; the total effect in this case includes not only the pregnancy itself, but also the treatments that coincide with pregnancy in our setting, including clinical care and management, and potentially culturally-specific responses to pregnancy, including stigma and adherence-related behaviors. Any total effect may in fact conceal heterogeneous mechanisms of effects. Some aspects of pregnancy (e.g., reduced adherence due to nausea, interruption or change of antiretroviral drug regimen) may increase risk of virologic failure, while others (e.g., findings that high levels of beta-estradiol may result in reduced viral loads [6], [47]) may decrease risk. A related factor, the specific choice of antiretroviral drugs for pregnant women, might increase or decrease risks of virologic failure. Future work should explore these different pathways of effect. In addition, future work should explore the effect of pregnancy on both clinical and immunological outcomes of HAART.

In this large study of clinical cohort data, we found that pregnancy after HAART initiation modestly increased both relative and absolute risks of virologic failure. While policy should never be based on a single study, these results provide more support [14]_ENREF_14 for the idea that family planning is an essential part of care and treatment for HIV-positive women in sub-Saharan Africa.

## Supporting Information

Table S1 **Main and sensitivity analyses for estimated effect of incident pregnancy on time to virologic failure among 5,494 women initiating HAART in South Africa, 2004–2009.**
(DOCX)

## References

[1] UNAIDS (2009). 2009 AIDS Epidemic Update.. http://dataunaidsorg/pub/Report/2009/2009_epidemic_update_enpdf.

[2] Pettifor AE, Rees HV, Kleinschmidt I, Steffenson AE, MacPhail C (2005). Young people's sexual health in South Africa: HIV prevalence and sexual behaviors from a nationally representative household survey.. Aids.

[3] UNAIDS/WHO (2007). AIDS Epidemic Update 2007.

[4] Black V, Hoffman RM, Sugar CA, Menon P, Venter F (2008). Safety and efficacy of initiating highly active antiretroviral therapy in an integrated antenatal and HIV clinic in Johannesburg, South Africa.. J Acquir Immune Defic Syndr.

[5] Homsy J, Bunnell R, Moore D, King R, Malamba S (2009). Reproductive intentions and outcomes among women on antiretroviral therapy in rural Uganda: a prospective cohort study.. PLoS ONE.

[6] Prins M, Meyer L, Hessol NA (2005). Sex and the course of HIV infection in the pre- and highly active antiretroviral therapy eras.. Aids.

[7] Guay LA, Musoke P, Fleming T, Bagenda D, Allen M (1999). Intrapartum and neonatal single-dose nevirapine compared with zidovudine for prevention of mother-to-child transmission of HIV-1 in Kampala, Uganda: HIVNET 012 randomised trial.. Lancet.

[8] Eshleman SH, Mracna M, Guay LA, Deseyve M, Cunningham S (2001). Selection and fading of resistance mutations in women and infants receiving nevirapine to prevent HIV-1 vertical transmission (HIVNET 012).. Aids.

[9] Jourdain G, Ngo-Giang-Huong N, Le Coeur S, Bowonwatanuwong C, Kantipong P (2004). Intrapartum exposure to nevirapine and subsequent maternal responses to nevirapine-based antiretroviral therapy.. N Engl J Med.

[10] Eshleman SH, Hoover DR, Chen S, Hudelson SE, Guay LA (2005). Nevirapine (NVP) resistance in women with HIV-1 subtype C, compared with subtypes A and D, after the administration of single-dose NVP.. J Infect Dis.

[11] Chi BH, Sinkala M, Stringer EM, Cantrell RA, Mtonga V (2007). Early clinical and immune response to NNRTI-based antiretroviral therapy among women with prior exposure to single-dose nevirapine.. Aids.

[12] Westreich D, Eron J, Behets F, Horst C, Van Rie A (2007). Survival in women exposed to single-dose nevirapine for prevention of mother-to-child transmission of HIV: a stochastic model.. J Infect Dis.

[13] Coffie PA, Ekouevi DK, Chaix ML, Tonwe-Gold B, Clarisse AB (2008). Maternal 12-month response to antiretroviral therapy following prevention of mother-to-child transmission of HIV type 1, Ivory Coast, 2003–2006.. Clin Infect Dis.

[14] Myer L, Carter RJ, Katyal M, Toro P, El-Sadr WM (2010). Impact of antiretroviral therapy on incidence of pregnancy among HIV-infected women in Sub-Saharan Africa: a cohort study.. PLoS Med.

[15] Kumar RM, Uduman SA, Khurrana AK (1997). Impact of pregnancy on maternal AIDS.. J Reprod Med.

[16] Burns DN, Landesman S, Minkoff H, Wright DJ, Waters D (1998). The influence of pregnancy on human immunodeficiency virus type 1 infection: antepartum and postpartum changes in human immunodeficiency virus type 1 viral load.. Am J Obstet Gynecol.

[17] French R, Brocklehurst P (1998). The effect of pregnancy on survival in women infected with HIV: a systematic review of the literature and meta-analysis.. Br J Obstet Gynaecol.

[18] Saada M, Le Chenadec J, Berrebi A, Bongain A, Delfraissy JF (2000). Pregnancy and progression to AIDS: results of the French prospective cohorts. SEROGEST and SEROCO Study Groups.. Aids.

[19] Lieve VP, Shafer LA, Mayanja BN, Whitworth JA, Grosskurth H (2007). Effect of pregnancy on HIV disease progression and survival among women in rural Uganda.. Trop Med Int Health.

[20] Minkoff H, Hershow R, Watts DH, Frederick M, Cheng I (2003). The relationship of pregnancy to human immunodeficiency virus disease progression.. Am J Obstet Gynecol.

[21] Tai JH, Udoji MA, Barkanic G, Byrne DW, Rebeiro PF (2007). Pregnancy and HIV Disease Progression during the Era of Highly Active Antiretroviral Therapy.. J Infect Dis.

[22] Keiser O, Gayet-Ageron A, Rudin C, Brinkhof MW, Gremlich E (2008). Antiretroviral treatment during pregnancy.. Aids.

[23] Melekhin VV, Shepherd BE, Stinnette SE, Rebeiro PF, Barkanic G (2009). Antiretroviral therapy initiation before, during, or after pregnancy in HIV-1-infected women: maternal virologic, immunologic, and clinical response.. PLoS ONE.

[24] Watts DH, Lu M, Thompson B, Tuomala RE, Meyer WA (2009). Treatment interruption after pregnancy: effects on disease progression and laboratory findings.. Infect Dis Obstet Gynecol.

[25] MacCarthy S, Laher F, Nduna M, Farlane L (2009). Responding to Her Question: A Review of the Influence of Pregnancy on HIV Disease Progression in the Context of Expanded Access to HAART in Sub-Saharan Africa.. AIDS Behavior.

[26] WHO (2006). Antiretroviral drugs and the prevention of mother-to-child transmission of HIV infection in resource-limited settings: Towards universal access.. http://www.who.int/hiv/pub/mtct/pmtct/.

[27] Mirochnick M, Capparelli E (2004). Pharmacokinetics of antiretrovirals in pregnant women.. Clin Pharmacokinet.

[28] Stek AM, Mirochnick M, Capparelli E, Best BM, Hu C (2006). Reduced lopinavir exposure during pregnancy.. Aids.

[29] Floridia M, Giuliano M, Palmisano L, Vella S (2008). Gender differences in the treatment of HIV infection.. Pharmacol Res.

[30] Aweeka F, Tierney C, Stek A, Sun X, Cohn S (2007). ACTG 5153s: Pharmacokinetic Exposure and Virological Response in HIV-1-infected Pregnant Women Treated with PI..

[31] Roustit M, Jlaiel M, Leclercq P, Stanke-Labesque F (2008). Pharmacokinetics and therapeutic drug monitoring of antiretrovirals in pregnant women.. Br J Clin Pharmacol.

[32] Zhang M, Huang Q, Huang Y, Wood O, Yuan W (2008). beta-Estradiol attenuates the anti-HIV-1 efficacy of Stavudine (D4T) in primary PBL.. Retrovirology.

[33] Sanne IM, Westreich D, Macphail AP, Rubel D, Majuba P (2009). Long term outcomes of antiretroviral therapy in a large HIV/AIDS care clinic in urban South Africa: a prospective cohort study.. J Int AIDS Soc.

[34] Westreich D, MacPhail P, Van Rie A, Malope-Kgokong B, Ive P (2009). Effect of pulmonary tuberculosis on mortality in patients receiving HAART.. Aids.

[35] Westreich DJ, Sanne I, Maskew M, Malope-Kgokong B, Conradie F (2009). Tuberculosis treatment and risk of stavudine substitution in first-line antiretroviral therapy.. Clin Infect Dis.

[36] Ray WA (2003). Evaluating medication effects outside of clinical trials: new-user designs.. Am J Epidemiol.

[37] Riddler SA, Haubrich R, DiRienzo AG, Peeples L, Powderly WG (2008). Class-sparing regimens for initial treatment of HIV-1 infection.. N Engl J Med.

[38] Hernán MA, Brumback B, Robins JM (2000). Marginal structural models to estimate the causal effect of zidovudine on the survival of HIV-positive men.. Epidemiology.

[39] Robins JM, Hernán MA, Brumback B (2000). Marginal structural models and causal inference in epidemiology.. Epidemiology.

[40] Westreich D, Cole SR, Tien PC, Chmiel JS, Kingsley L (2010). Time scale and adjusted survival curves for marginal structural cox models.. Am J Epidemiol.

[41] Hernán MA, Robins JM (2006). Estimating causal effects from epidemiological data.. J Epidemiol Community Health.

[42] Cole SR, Hernan MA (2008). Constructing inverse probability weights for marginal structural models.. Am J Epidemiol.

[43] Westreich D, Cole SR (2010). Invited commentary: positivity in practice.. Am J Epidemiol.

[44] Sha BE, Tierney C, Cohn SE, Sun X, Coombs RW (2011). Postpartum viral load rebound in HIV-1-infected women treated with highly active antiretroviral therapy: AIDS Clinical Trials Group Protocol A5150.. HIV Clin Trials.

[45] Cole SR, Hernán MA, Robins JM, Anastos K, Chmiel J (2003). Effect of highly active antiretroviral therapy on time to acquired immunodeficiency syndrome or death using marginal structural models.. Am J Epidemiol.

[46] Petersen ML, Wang Y, van der Laan MJ, Bangsberg DR (2006). Assessing the effectiveness of antiretroviral adherence interventions. Using marginal structural models to replicate the findings of randomized controlled trials.. J Acquir Immune Defic Syndr.

[47] Gandhi M, Bacchetti P, Miotti P, Quinn TC, Veronese F (2002). Does patient sex affect human immunodeficiency virus levels?. Clin Infect Dis.

