# Supplementary material for: Pregnancy and Virologic Response to Antiretroviral Therapy in South Africa
Source: PLoS One. 2011 Aug 2;6(8):e22778. doi: 10.1371/journal.pone.0022778 (PMC3149058; doi:10.1371/journal.pone.0022778)
Supplement: Table S1 — Main and sensitivity analyses for estimated effect of incident pregnancy on time to virologic failure among 5,494 women initiating HAART in South Africa, 2004–2009. (DOCX) [file pone.0022778.s001.docx]

Table S1. Main and sensitivity analyses for estimated effect of incident pregnancy on time to virologic failure among 5,494 women initiating HAART in South Africa, 2004-2009.

|  |  | HR | 95% CL |
| --- | --- | --- | --- |
|  | Main analysis | 1.34 | 1.02, 1.78 |
|  | |  |  |
| 1 | Population: restrict to initial virologic success | 1.39 | 0.97, 1.99 |
| 2 | Population: extra prevalent exclusions | 1.33 | 1.00, 1.77 |
| 3 | Outcome: failure/mortality | 1.25 | 0.96, 1.62 |
| 4 | Outcome: failure/mortality/drop-out | 0.96 | 0.78, 1.17 |
| 5 | Outcome: confirmed virologic failures only | 1.43 | 0.80, 2.55 |
| 6 | Population, outcome: combining analyses 1 and 5 | 1.27 | 0.57, 2.84 |
| 7 | Exposure: prevalent and incident | 1.12 | 0.86, 1.46 |
| 8 | Analysis: multiple imputations | 1.27 | 0.98, 1.66 |
| 9 | Analysis: dropping carry-forward | 1.25 | 0.91, 1.73 |
